# Accessible Surfaces of Beta Proteins Increase with Increasing Protein Molecular Mass More Rapidly than Those of Other Proteins

**DOI:** 10.1371/journal.pone.0028464

**Published:** 2011-12-01

**Authors:** Anna V. Glyakina, Natalya S. Bogatyreva, Oxana V. Galzitskaya

**Affiliations:** 1 Institute of Protein Research, Russian Academy of Sciences, Pushchino, Moscow Region, Russia; 2 Institute of Mathematical Problems of Biology, Russian Academy of Sciences, Pushchino, Moscow Region, Russia; University of South Florida College of Medicine, United States of America

## Abstract

Here we present a systematic analysis of accessible surface areas and hydrogen bonds of 2554 globular proteins from four structural classes (all-α, all-β, α/β and α+β proteins) that is aimed to learn in which structural class the accessible surface area increases with increasing protein molecular mass more rapidly than in other classes, and what structural peculiarities are responsible for this effect. The beta structural class of proteins was found to be the leader, with the following possible explanations of this fact. First, in beta structural proteins, the fraction of residues not included in the regular secondary structure is the largest, and second, the accessible surface area of packaged elements of the beta-structure increases more rapidly with increasing molecular mass in comparison with the alpha-structure. Moreover, in the beta structure, the probability of formation of backbone hydrogen bonds is higher than that in the alpha helix for all residues of α+β proteins (the average probability is 0.73±0.01 for the beta-structure and 0.60±0.01 for the alpha-structure without proline) and α/β proteins, except for asparagine, aspartic acid, glycine, threonine, and serine (0.70±0.01 for the beta-structure and 0.60±0.01 for the alpha-structure without the proline residue). There is a linear relationship between the number of hydrogen bonds and the number of amino acid residues in the protein (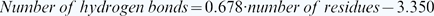).

## Introduction

Analysis of the accessible surface area (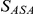) is a necessary element in studying protein-protein interactions and the process of protein folding. The technique of quantitative protein surface analysis using high-resolution X-ray data was first proposed by Lee and Richards [1] who analyzed the accessible surface area (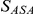). In particular, using high-resolution X-ray data on 37 monomeric globular proteins with molecular masses (M) of 4-35 kDa it has been shown [2] that the dependence of 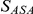 on *M* is a power law with an extent of 0.73. For oligomeric proteins, this value was found to be 0.76 [3]. It has been demonstrated that such a dependence results from the peculiarities of the protein surface relief [4], [5]. The aim of this work is to elucidate the features of these peculiarities for different “architectural” classes of proteins. In this study we addressed two questions: (i) what is the relationship between molecular mass and the accessible surface area of proteins from the four general structural classes, and (ii) how much the accessible surfaces vary in molecular mass, shape, and structural type.

The deviation of the power law extent from 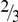 in the 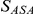 — *M* dependence was considered as an indication of the protein surface fractal structure [6], [7]. Strictly speaking, a surface is fractal if the dependence of the minimal number of probe bodies (balls, cubes, etc.) fully covering the surface on the probe size is a power law:
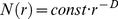
(1)with the extent 2<*D<*3 not coinciding with the topological dimension *(D_top_  = *2) and *D* being a fractal dimension [8]. The strict fractal dimension is determined at *r* →0. For self-similar bodies, the relationship between the fractal surface area and the value of confined volume *(V)* has the following power law [9]:
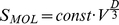
(2)


Qualitatively at *D>2* this means that the size of irregularities increases with the increasing particle size. The use of both dependencies was justified by the observation that the extent of protein asphericity did not depend on the protein molecular mass [4]. The observed [2] more rapid increase of the protein accessible surface area 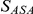 with the increasing molecular mass, *(M)* as compared to that of isometric particles, can be explained by the increase of the protein surface area caused by the increasing protein size. As a result, two levels of the protein surface structural organization have been detected: fractal on a small-scale level (2–7 Å) and block-like on a large-scale level [5]. Large-scale surface defects are revealed on macroscale, which is interpreted as a result of packing of secondary structure elements.


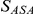
 is not just a geometric measure. It is also of physical significance: the extent of the gain in hydrophobic energy is proportional to the extent of reduction in 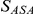 [10]. For this reason, 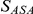 has become an important factor in the analyses of protein folding and protein-protein interactions [11]. It has been shown that structural determinants, taking into account both the protein shape and its size, show good agreement with experimentally observed rates of protein folding [12]. Moreover, a relationship has been established between the protein accessible surface area and the number of native contacts in its structure [13]. Hidden dependencies between protein structural class specific fractal dimension magnitudes and kinetic–thermodynamic parameters (folding/unfolding rate, folding/unfolding free energy) were studied not long ago [14]–[16]. The results of this study confirmed the dependence of fractal dimension values on the fold type and on the location and connectivity of the secondary structures.

Here we offer a systematic analysis of accessible surface areas and hydrogen bonds of 2554 globular proteins with high resolution to answer the questions as to what structural class demonstrates the most rapid growth of the protein accessible surface area with concurrently increasing protein molecular mass and what structural peculiarities are responsible for such a behavior. It has been shown that accessible surface areas of proteins from the beta-structural class increase with the increasing molecular mass more rapidly than those of other classes. We have found two possible reasons for this fact: (i) all-β proteins have more amino acid residues in the irregular structure than proteins from other classes, and (ii) accessible surface areas of packaged elements of the beta-structure increase more rapidly with the increasing molecular mass than those of the alpha-structure. Moreover, the probability of formation of backbone hydrogen bonds in the beta structure is higher than in alpha helix for practically all amino acid residues except for proline, while aspartic acid and threonine have practically equal probabilities for the two considered structures.

## Materials and Methods

### Preprocessing of data

We selected single-domain proteins with resolution higher than 3 Å and well-refined crystal structures, with less than 25% sequence identity belonging to classes a, b, c, and d (according to the SCOP classification, release 1.65) [17]. The obtained dataset includes **2554** proteins (see Tables 1, 2): 499 proteins from class a (all-α proteins), 656 proteins from class b (all-β proteins), 709 proteins from class c (α/β proteins), and 690 proteins from class d (α+β proteins). For selection, the general criterion was the absence of unresolved (disordered) residues.

**Table 1:** Structural characteristics of 1155 globular protein domains from classes α and β.

	Class α (499 proteins)				Class β (656 proteins)			
Range of length	Number of proteins (average length of protein)	Molecularsurface S, Å^2^	Accessiblesurface S, Å^2^	Molecularmass	Number of proteins (average length of protein)	Molecularsurface S, Å^2^	Accessiblesurface S, Å^2^	Molecularmass
51–100	170 (78)	4633±63	5352±74	8853±119	181 (82)	4445±54	5133±62	9076±111
101–150	192 (127)	6897±67	7624±85	14338±123	245 (121)	6091±53	6754±60	13363±105
151–200	77 (171)	8713±117	9383±142	19174±214	127 (173)	8247±97	8831±106	19202±157
201–250	22 (223)	10869±241	11320±287	25356±389	47 (218)	10138±186	10695±240	24026±223
251–300	21 (278)	12834±380	13131±414	31161±495	33 (273)	12137±234	12427±281	30214±306
301–350	17 (324)	14454±347	14352±339	36615±510	23 (326)	13632±319	13679±353	35701±429

**Table 2:** Structural characteristics of 1399 globular protein domains from classes α/β and α+β.

	Class α/β (709 proteins)				Class α+β (690 proteins)			
Range of length	Number of proteins (average length of protein)	Molecular surface S, Å^2^	Accessible surface S, Å^2^	Molecular mass	Number of proteins (average length of protein)	Molecular surface S, Å^2^	Accessible surface S, Å^2^	Molecular mass
51–100	22 (89)	4650±76	5286±90	9850±172	161 (81)	4543±57	5204±63	9060±119
101–150	124 (128)	6312±76	6894±86	14085±145	246 (126)	6584±58	7265±68	14042±103
151–200	194 (174)	8063±55	8541±60	19132±122	132 (174)	8530±98	9126±116	19416±147
201–250	147 (225)	10183±90	10509±101	24795±145	86 (222)	10204±103	10586±122	24586±184
251–300	123 (274)	11700±102	11757±115	30106±181	39 (272)	12063±204	12241±240	30622±309
301–350	99 (325)	13345±127	13170±134	35771±214	26 (322)	14236±276	14171±282	36549±374

Simultaneously, we considered the re-refined structures to demonstrate that the dependence between accessible surfaces and molecular masses described in this work was not significantly altered. The re-refined structure models were taken from the PDB_REDO databank (http://www.cmbi.ru.nl/pdb_redo/) [18]. We selected the protein structures in accordance with our dataset. As a result, we obtained **1498** structures: 284 proteins from class a (all-α proteins), 398 proteins from class b (all-β proteins), 427 proteins from class c (α/β proteins), and 389 proteins from class d (α+β proteins).

### Calculation of accessible surface area and protein molecular surface

We calculated the accessible surface area 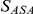 for each protein considered. The calculations were made with the YASARA program [http://yasara.org] using 1.4 Å as the probe radius of a water molecule. The difference between the molecular surface and the accessible surface is that the accessible surface area (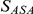) is a surface formed by the center of a probe molecule rolled over a protein molecule, while the molecular surface is a surface formed by the Van der Waals sphere of a probe molecule rolled over a protein molecule. If the probe is water, a water molecule is modeled by a sphere of radius 1.4 Å. This means that the molecular surface is “thinner” than 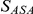 and the “distance” between them is 1.4 Å. In fact, the molecular surface is obtained from the Van der Waals surface if all crevices and interiors inaccessible for water are smoothed by means of the Van der Waals surface of the water molecule.

### Hydrogen bonds observed in spatial structures of proteins

Hydrogen bonds were searched for in the same dataset. We collected statistics separately for two variants of hydrogen bonds. In the first case, backbone hydrogen bonds (that is, hydrogen bonds where the donor is an NH-group of the protein backbone and the acceptor is an O-atom of the protein backbone) were analyzed with the standard DSSP program [19]. For each NH-group, only one hydrogen bond (which had the best energy, according to DSSP) was taken into consideration in this case. The criterion of hydrogen bond formation was that recommended by the DSSP authors (the calculated energy lower than -0.5 kcal/mol). In the other case, we calculated the hydrogen bonds taking into account both backbone and side-chains (that is, hydrogen bonds where the donor and acceptor belong to the protein side-chain). For this purpose we used the YASARA program. The criterion of hydrogen bond formation was that recommended by the YASARA authors (the calculated energy lower than −1.5 kcal/mol).

During the calculation, the hydrogen bonds were "ascribed" to acceptor residues according to the type of structure (helical-structure or beta-structure), which resulted in two sets of probability values for each type of amino acid residues. Along with the DSSP program, the helical-structure includes residues from α- and 3_10_–helices. The beta-structure includes residues from isolated β-bridges and extended strands involved in β-sheets. Residues from π-helices, hydrogen-bonded turns and bends are included in the irregular structure (coil). The probability of hydrogen bond formation was calculated as the total number of hydrogen bonds of the corresponding variant (backbone−backbone for helical-structure and backbone−backbone for beta-structure) formed by each type of amino acid residues divided by the total number of residues of this type in the considered secondary structure in the dataset.

### Error estimation

**Figure 1:**
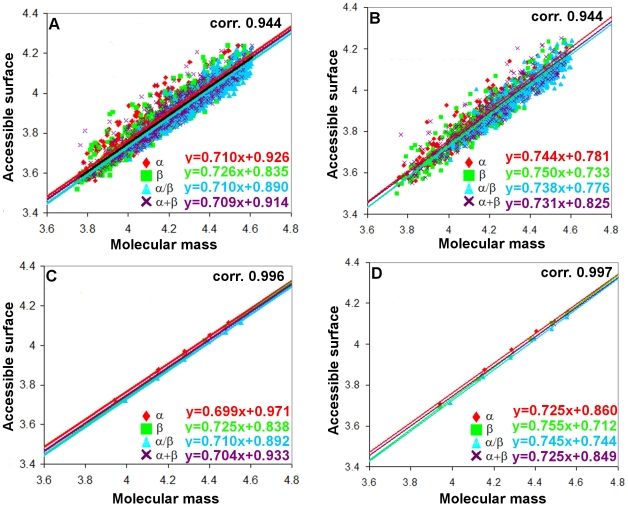
Log-log dependences of accessible surface areas on protein molecular masses for four structural classes of proteins. Cases (A) and (C) for general dataset of proteins and cases (B) and (D) for re-refined protein structures. In cases (A) and (B), values for all proteins without averaging (the number of points corresponds to the number of proteins in each structural class) were considered. And in cases (C) and (D) these values were averaged in the given region of the protein lengths (six points for each structural class).

The standard deviation for the slopes of the straight lines (see Figure 1) of the log-log dependences of the accessible and molecular surface areas versus the protein molecular masses is calculated as 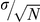, where *N* is the number of proteins and σ is the root-mean-square deviation:
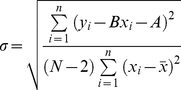
(3)where 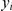 is the molecular (or accessible) surface, 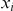 is the molecular mass, and 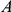 and 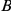 are coefficients of the linear equation 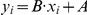. Standard deviations for these values are in the third decimal place.

## Results and Discussion

### Accessible surfaces in four structural classes

For 2554 globular proteins belonging to four structural classes according to the SCOP classification we calculated accessible surface areas and molecular masses (see Tables 1, 2, Figure 1A,C). The slopes of the straight lines (the tangents of the slope) of the log-log dependences of the accessible surface areas (with account of hydrogen atoms) on the protein molecular masses are given for the four structural classes in Table 3. The slopes of the lines were obtained for two cases: first, when considering all proteins, and second, when averaging was made in the specified region of the length of proteins, which gave six points. One can see that the beta structural class of proteins has a larger power in the analyzed dependences for surface areas than other structural classes. The same trend was observed when considering the re-refined protein structures (see Table 3 and Figure 1B,D). The higher value of the fractal dimension from the 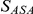 *— M* dependence can be interpreted as an increase of the number of large-scale irregularities on the protein surface with an increase of the protein size [5]. The packing of secondary structure elements is important for the observed protein surface properties. It would be of interest to learn how such packing of secondary structure elements influences the irregularities of the protein surface.

**Table 3:** Slopes of straight lines of log-log dependences of accessible surface areas on protein molecular masses for two databases: PDB (2554 proteins) and PDB_REDO (1498 proteins).

	PDB	REDO_PDB	PDB	REDO_PDB
Class	(number of points)	(number of points)	(number of points)	(number of points)
a	0.710 (499)	0.744 (284)	0.699 (6)	0.725 (6)
b	0.726 (656)	0.750 (398)	0.725 (6)	0.755 (6)
c	0.710 (709)	0.738 (427)	0.710 (6)	0.745 (6)
d	0.709 (690)	0.731 (389)	0.704 (6)	0.725 (6)

**Figure 2:**
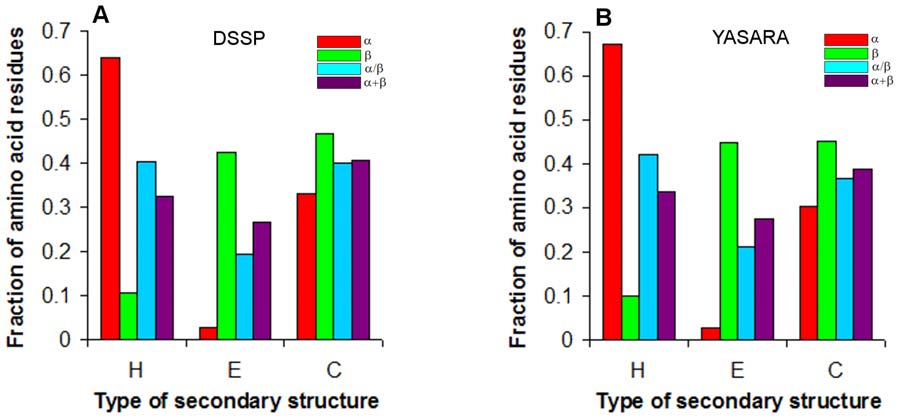
Fraction of amino acid residues of each type of secondary structure. H, helix (α and 3_10_); E, β structure; C, coil for four structural classes of proteins calculated using the DSSP (A) and YASARA (B) programs.

To find the structural peculiarities responsible for the above, we constructed the statistics of occurrence of residues in three different structural classes (alpha-helix, beta-structure, and coil). Since different programs make different assignments of secondary structures, we used two programs for this purpose: DSSP and YASARA. It turned out that the secondary structure assignments obtained with these programs are practically the same. An interesting result obtained from the statistics is that the fraction of residues involved in the regular secondary structure is larger for all-α proteins and the least for all-β proteins according to the both programs used (see Figure 2). Such a difference can be explained by the existence of the largest number of residues in the coil conformation on the surfaces of beta structural proteins.

The higher value of fractal dimension from the 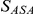 *— M* dependence for all-β proteins can be explained by at least two reasons: first, by a large fraction of residues in the loop regions, and second, by the fact that the accessible surface area of packaged elements of the beta-structure increases more rapidly with the increasing molecular mass than that of the alpha-structure.

**Figure 3:**
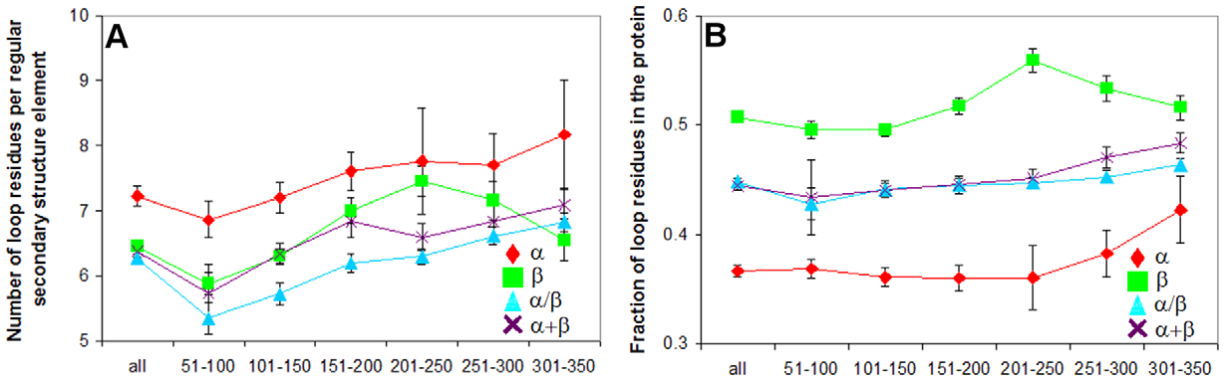
Number of loop residues per regular secondary structure element (A) and the fraction of loop residues in the protein structure (B) in the given region of amino acid residues in four structural classes of proteins.

To clarify this situation, we made an additional analysis of protein structures from our dataset. Two parameters were considered: (i) the number of loop residues per regular secondary structure element (Figure 3A), and (ii) the fraction of loop residues in the protein structure (Figure 3B). As seen, for all considered sequence sections, the former is higher in all-α proteins, while the latter is higher in all-β proteins. With a given value of parameter (i) or (ii), the dependence between the accessible surface area and the protein molecular mass allows assessing the fractal dimension of helical and beta-structural surfaces and an increase/decrease of this dependence with increasing/ decreasing parameter (i) or (ii).

**Figure 4:**
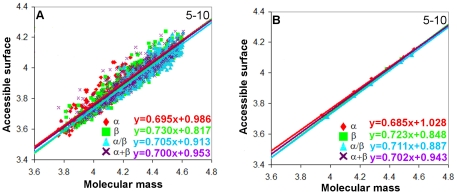
Log-log dependences of accessible surface areas on protein molecular masses for four structural classes of proteins where the number of loop residues per regular secondary structure element varies from 5 to 10. In case (A), values for all proteins without averaging were considered (the number of points corresponds to the number of proteins in each structural class). And in case (B) these values were averaged in the given region of protein lengths (six points for each structural class).

With parameter (i) covering range1-5, we do not have a sufficient number of proteins for the statistical analysis, unlike range 5–10 where we have 311 proteins from class a, 448 proteins from class b, 543 proteins from class c, and 452 proteins from class d. For the β proteins, transition from the 1–5 to 5–10 residues results in an increase of the power of dependence of the accessible surface on molecular mass from 0.709 to 0.730 (see Figure 4, range 5–10, range 1–5 is not shown). Thus, over range 5–10, the accessible surface areas of the beta-structure grow with the increasing molecular mass more rapidly than those of the alpha-structure (0.730 against 0.695 for accessible surfaces).

**Figure 5:**
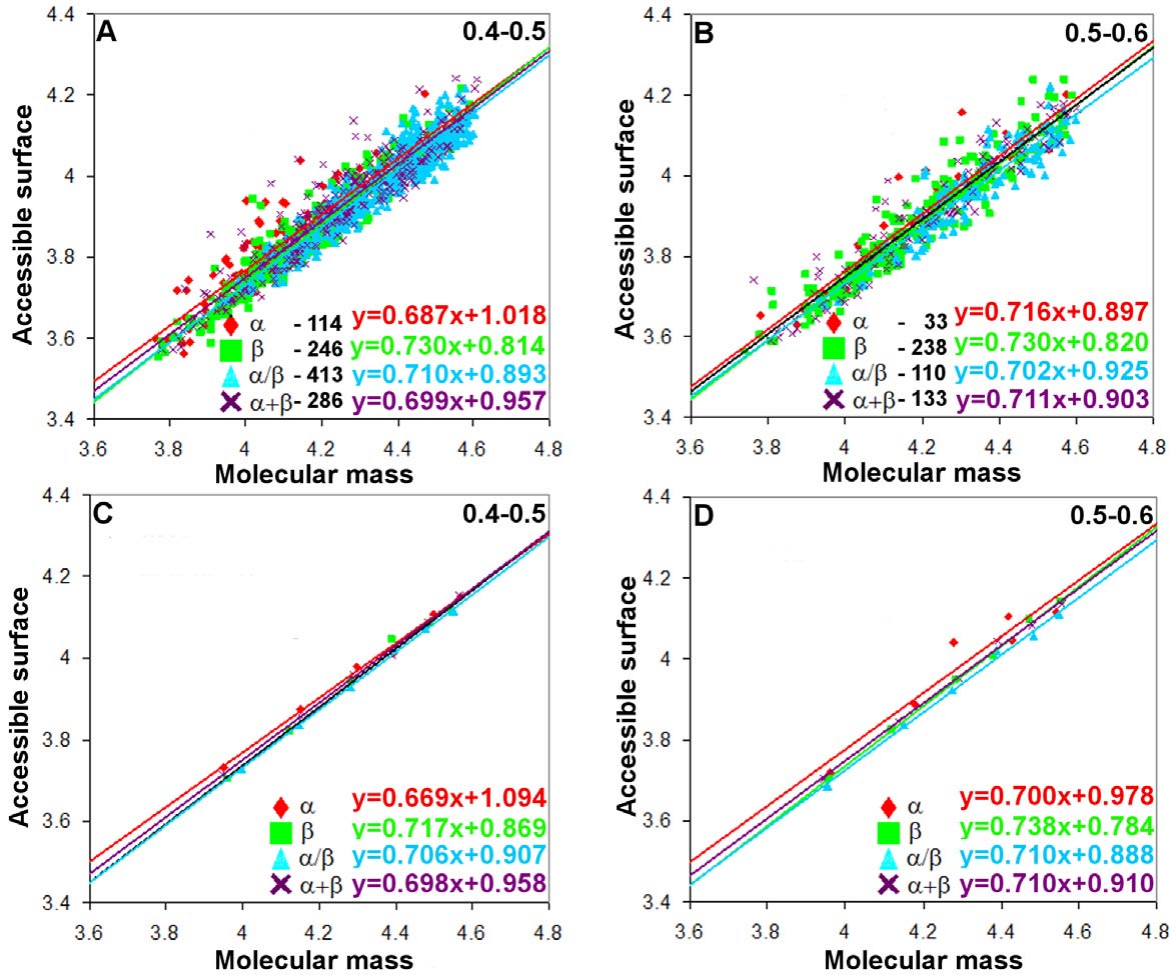
Log-log dependences of accessible surface areas on protein molecular masses for four structural classes of proteins where the fraction of loop residues in the protein structure is as follows: (A, C) 0.4–0.5; (B, D) 0.5–0.6. In cases (A) and (B), values for all proteins without averaging were considered (the number of points corresponds to the number of proteins in each structural class). And in cases (C) and (D) these values were averaged in the given region of protein lengths (six points for each structural class).

As for parameter (ii), with fractions 0.4–0.5 and 0.5–0.6, we have a sufficient number of proteins for the statistical analysis in all the four structural classes (with exception for class a where in fraction 0.5–0.6 covering chain lengths 201–250 and 251–300 there is only one protein). For the given value of parameter (ii), the beta structural class of proteins has a larger power of dependences for both surface areas than alpha helical proteins. This means that accessible and molecular surface areas of the beta-structure increase with the increasing molecular mass more rapidly than those of the alpha-structure. Construction of two such dependences with different numbers of loop residues in the four structural protein classes allows us to conclude that an increase in the length of loops results in the increasing 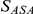 value in monomeric proteins of different structural classes. The dependence of the accessible surface area on molecular mass for all-β proteins increases from 0.717 to 0.738 (from 0.669 to 0.700 for all-α proteins) (Figure 5C,D).

**Figure 6:**
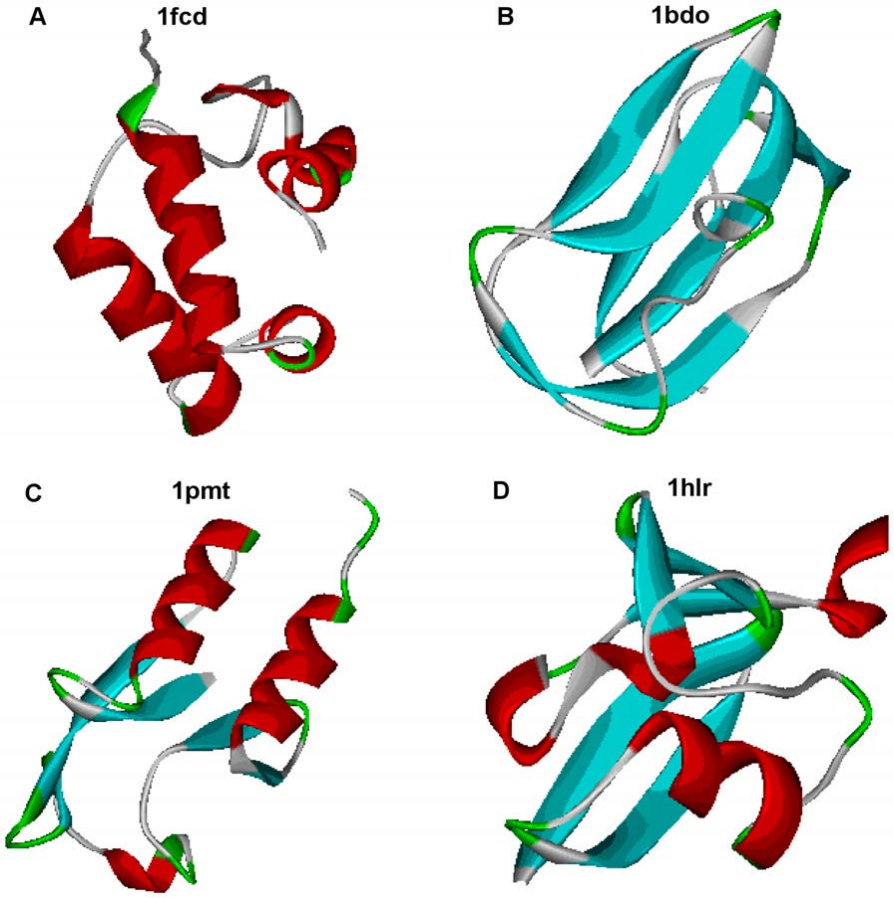
Protein structures from four general structural classes with the same length of proteins (80 amino acid residues) and the same fraction of residues in loop region (0.55).

Figure 6 demonstrates the protein structures from four general classes with the same length of proteins and the same fraction of residues in the loop region. But the number of loop residues per regular element of the secondary structure is different especially for α structural proteins.

Thus, it can be concluded that for all-β proteins at least two factors (surface area of packaged elements of the beta-structure increases with the increasing molecular mass more rapidly than that of alpha-helix and a larger number of loop residues in all-β proteins) upregulate the power in the 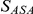 *— M* dependence.

### Hydrogen bonds in spatial structures of proteins

**Figure 7:**
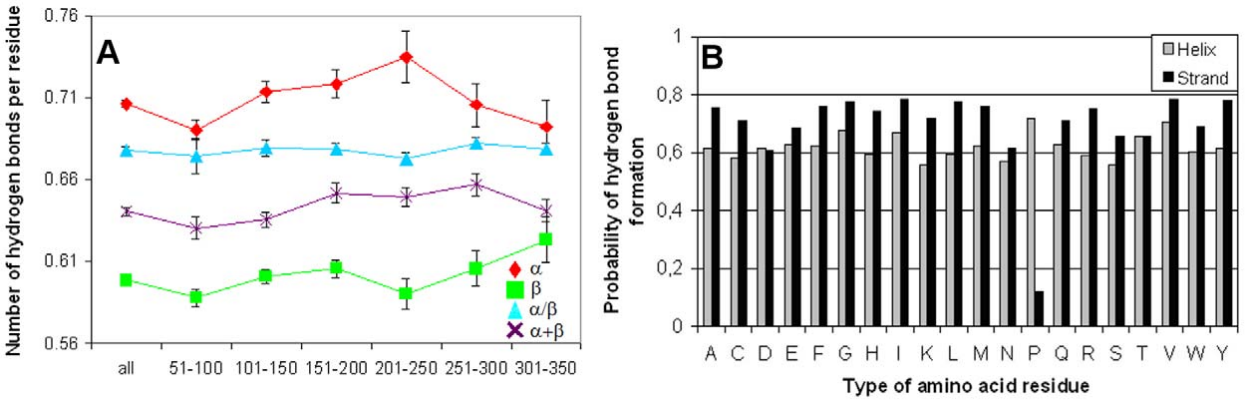
Distribution of hydrogen bonds per residue in the given region of a number of amino acid residues in four structural classes of proteins calculated with DSSP (A) and probability of formation of backbone hydrogen bonds (B). Hydrogen bonds are assigned to acceptor residues. Black bars correspond to the hydrogen bonds in beta structure, gray bars to the helical structure. The average probability of hydrogen bond formation for helical-structure is 0.62±0.01 (0.62±0.01 without proline) and for beta-structure 0.69±0.03 (0.72±0.01 without proline).

**Figure 8:**
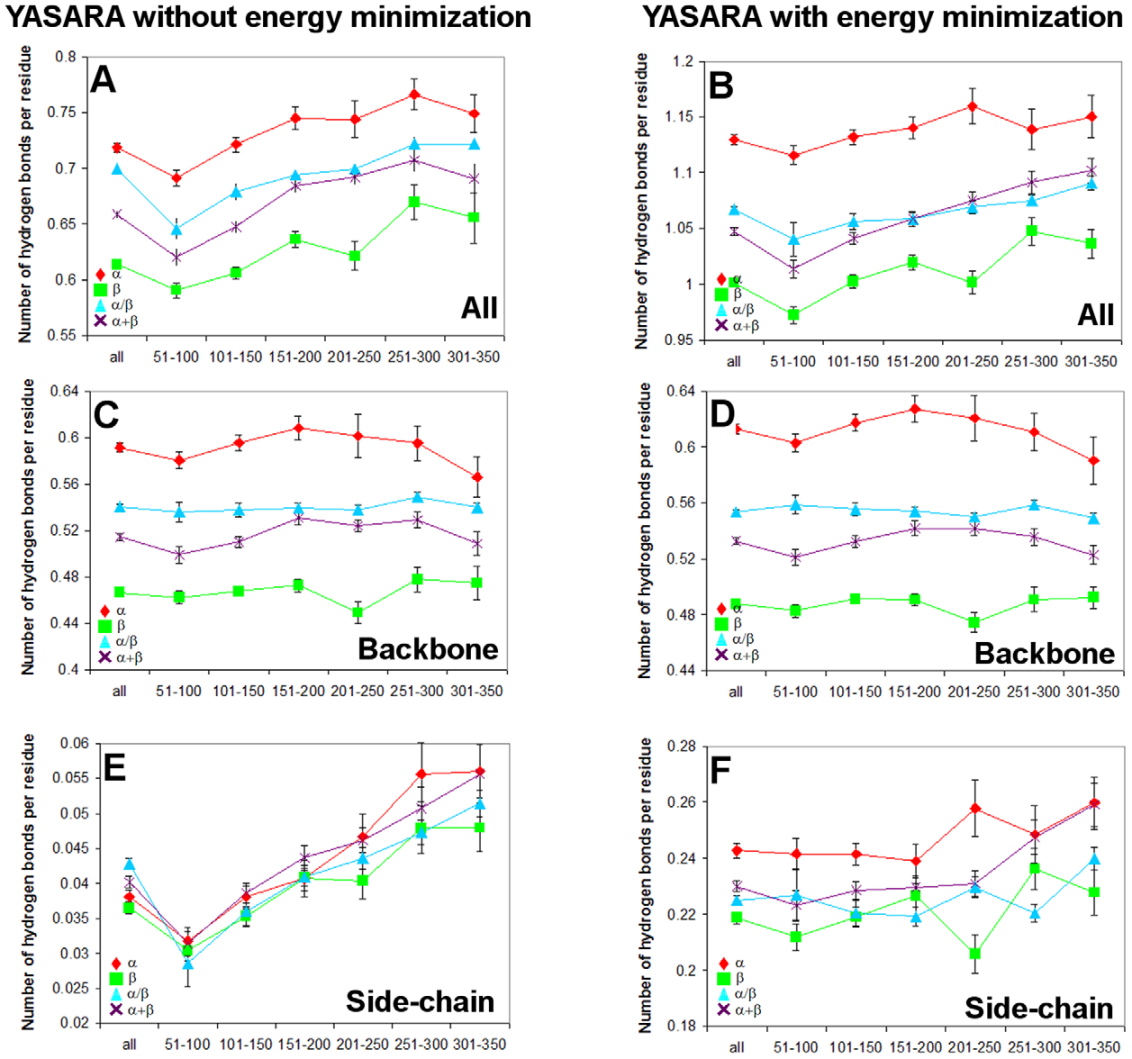
Distribution of hydrogen bonds per residue in the given region of amino acid residues in four structural classes of proteins calculated with YASARA. Cases (A, C, E) without and (B, D, F) with energy minimization.

Since the total number of hydrogen bonds is proportional to the protein helix and sheet content we calculated the number of hydrogen bonds per residue in each structural class of proteins. The distribution of hydrogen bonds per residue in the given range of protein chain lengths determined with the DSSP program is shown in Figure 7A. As seen, alpha structural proteins have more hydrogen bonds per residue, which agrees with the fact that this class of proteins has the largest number of residues in the regular structure. Using the DSSP program we can consider only backbone hydrogen bonds. For analysis of all possible hydrogen bonds in the proteins we used another program, YASARA, which was also applied to calculate accessible and molecular surface areas. In this case we obtained similar patterns of hydrogen bonds per residue in different classes (Figure 8).

Although for α/β proteins the fraction of residues in the coil state is larger than that for all-α proteins (according to the DSSP and YASARA programs), the number of hydrogen bonds per residue for these two classes of proteins is practically the same (see Figure 8A). The number of hydrogen bonds depends on the protein size, and this dependence is crucial since consideration of all proteins taken together (i.e., without regard to their size) changes the result dramatically, namely: α/β proteins have the same number of hydrogen bonds per residue (Figure 8A), while actually they are in the middle of the averaged values among the four classes, as judged by the analysis using different window sizes (Figure 8A). This situation is a result of a different number of proteins in each size range. Therefore, the average value over six regions does not necessarily coincide with the average over all proteins without dividing them into regions. One can see that the difference between the fractions of irregular structure residues is the largest, about 15%, but the difference in the number of hydrogen bonds per residue is not so great. One of possible explanations of this fact can be a different contribution of side chains or different saturation of hydrogen bonds in alpha helices and beta structures or both.

To check the first assumption, we analyzed the number of hydrogen bonds per residue in each structural class separately for the backbone and side-chains within a given size range where the average length of proteins is nearly the same in each structural class (Figures 8C, E). One can see that the backbone dependence is similar to that for all hydrogen bonds, and the contribution of side chains is insignificant. An advantage of the YASARA program is a possibility to perform energy minimization of protein structures and to check the number of hydrogen bonds after this procedure. A fascinating result that we obtained is the increasing number of hydrogen bonds per residue after minimization (Figure 8B). And the distribution of hydrogen bonds per residue in the given region of a number of amino acid residues in protein chains is similar to that after using these programs without energy minimization. In this case we checked once again the contribution of the backbone and side-chains in the formation of hydrogen bonds and found that the contribution of side-chains increased more than the contribution of the backbone hydrogen bonds after energy minimization (Figure 8F). Before energy minimization the contribution of side-chain hydrogen bonds was very small in comparison with that of backbone hydrogen bonds. The reason is that side-chains have no alternative donors and acceptors from water molecules, and all hydrogen bonds are formed by atoms from side-chains and the backbone. We have calculated that the accessible surfaces and volumes of structures decrease after energy minimization by 5% and 1%, respectively.

**Figure 9:**
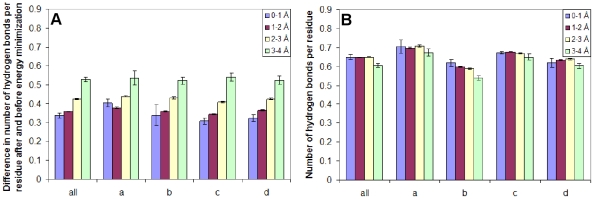
Average number of hydrogen bonds per residue for proteins with different resolutions. (A) Difference in the number of hydrogen bonds per residue after and before energy minimization (YASARA). (B) Number of hydrogen bonds per residue (DSSP).

We constructed the difference between the number of hydrogen bonds per residue before and after energy minimization according to our division into four groups for X-ray structures (0-1, 1–2, 2–3, and 3–4 Å resolution). It was found that the lower the resolution, the larger the number of hydrogen bonds gained by YASARA. As concerns the DSSP program, we did not obtain such an effect, except for proteins with resolution higher than 3 Å which have been deleted from our dataset (see Figure 9).

**Figure 10:**
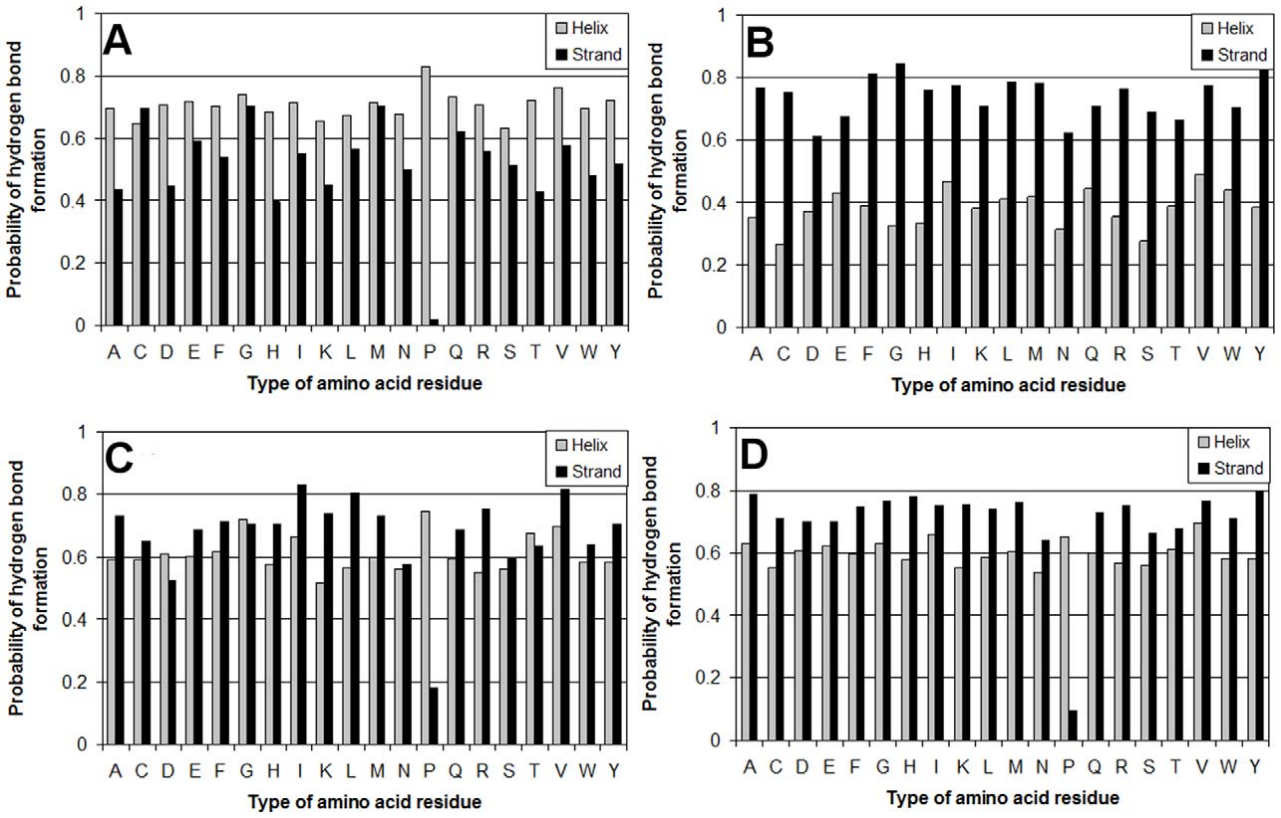
Statistics of hydrogen bonds observed in protein spatial structures. Probability of formation of backbone hydrogen bonds for four structural classes of proteins: (A) class a; (B) class b; (C) class c and (D) class d.

**Figure 11:**
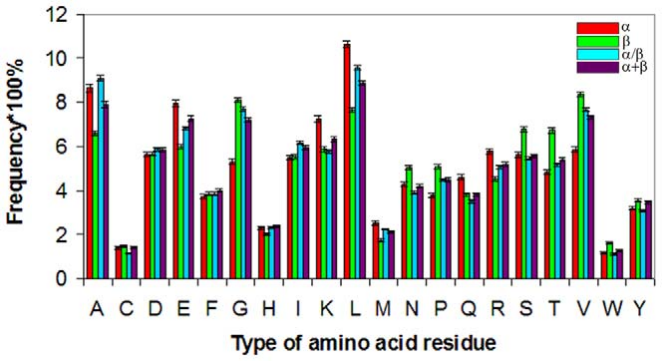
Average frequency of occurrence of each type of amino acid residue in four structural classes of proteins.

To verify the other assumption for different saturation of hydrogen bonds in different structures, we constructed the statistics of hydrogen bonds for each of the 20 types of amino acid residues in two structural classes: helical-structures and beta-structures according to the DSSP program. The statistics of hydrogen bonds was analyzed using the same dataset of 2554 three-dimensional protein structures. We searched for two separate variants of hydrogen bonds: backbone−backbone (both the donor and the acceptor are in the protein backbone and hydrogen bonds belong to the helical-structure) and backbone−backbone (both the donor and the acceptor are in the protein backbone and hydrogen bonds belong to the beta-structure). Then, the probabilities of formation (by each type of amino acid residues) of hydrogen bonds of a given variant were calculated. During the calculation, the hydrogen bonds were "ascribed" to acceptor residues, resulting in a set of probability values for each type of amino acid residues. Figures 7B, 10 show the obtained probabilities of formation of hydrogen bonds of different variants for each of the 20 types of amino acid residues. An interesting result of this analysis is that the saturation of hydrogen bonds is higher in the beta-structure than in the helical-structure. Practically for all amino acid residues the probability of formation of hydrogen bonds in the beta-structure is higher than in the helical-structure, that is, the saturation is stronger for the beta-structure, with one exception for proline, while aspartic acid and threonine have practically equal probabilities for the two considered structures. It should be underlined that the number of threonine residues occurring in the four classes of proteins is larger for all-β proteins than for other protein structures, and the number of aspartic acid residues is practically the same in the four classes (see Figure 11). The average probability of hydrogen bond formation for the helical-structure is 0.62±0.01 (0.62±0.01 without proline), and for the beta-structure it is 0.69±0.03 (0.72±0.01 without proline).

One can expect that the differences in saturation of hydrogen bonds for the alpha and beta-structures would arise from the edge effects, that is, from the differences between the average numbers of residues in the edge strands of beta-sheets and the helical ends. The DSSP program assigns a “strand” to residues in middle strands if both backbone atoms are H-bonded. So middle strands will always be fully saturated. Edge strands should be half-saturated only. Helices must have H-bonds for the both backbone atoms, the first and last turns of a helix should be half-saturated only. For all-α proteins the number of loop residues per regular secondary structure element is higher over all considered ranges of protein lengths than that for other classes of proteins (see Figure 3A). Compared to an all-α protein of the same size, an all-β protein in general would have more secondary structure elements (beta-strands), hence more loops and turns, but it would have fewer secondary structure blocks (beta-sheets), hence higher saturation of hydrogen bonds in these blocks. More clearly this effect is seen for the d (α+β proteins with segregated alpha and beta regions) and c class proteins (α/β proteins with mixed alpha and beta structures). As for the average probability of hydrogen bond formation for each of the four classes, for the beta-structure this probability is higher for all residues from class d, but in class c asparagine, aspartic acid, glycine, serine, and threonine have higher or equal probability of alpha-helix formation as compared with the beta-structure (see Figure 10C,D).

**Figure 12:**
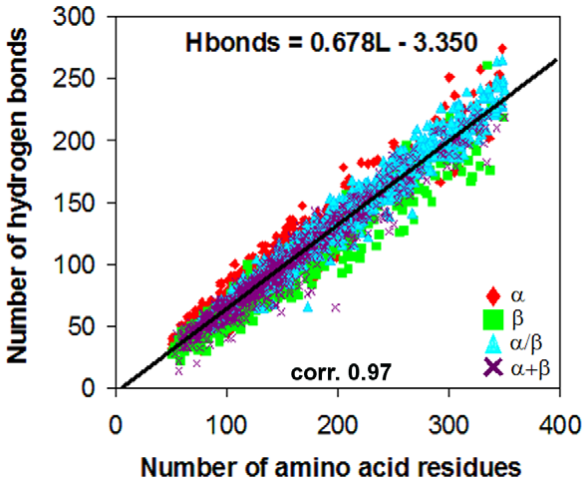
Dependences of a number of hydrogen bonds on the number of amino acid residues in protein.

Since the total number of hydrogen bonds is proportional to the protein helix and sheet content, Stickle et al. [20] suggested an equation for estimation of hydrogen bonds in proteins (their dataset consisted of 42 X-ray structures of proteins): 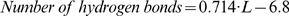, where *L* is the number of residues. We suggested close coefficients after studying 2554 structures: 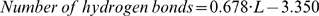 (Figure 12). Correlation coefficient is 0.97.

The results of our analysis of protein surfaces and its detailed structure allow us to obtain important information on protein structures: the probability of formation of backbone hydrogen bonds of the beta structure is higher than in alpha helix practically for all amino acid residues with one exception for proline.

Consideration of two additional parameters (the number of loop residues per regular secondary structure element and the fraction of loop residues in the protein structure) showed that for all-β proteins at least two factors (accessible and molecular surface areas of packaged elements of the beta-structure increase with the increasing molecular mass more rapidly than those of alpha-helix and a larger number of loop residues in all-β proteins) upregulate the power of the 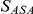 *— M* dependence.
